# Changes in Grandparental Childcare During the Pandemic and Mental Health: Evidence From England

**DOI:** 10.1093/geronb/gbac104

**Published:** 2022-09-19

**Authors:** Giorgio Di Gessa, Valeria Bordone, Bruno Arpino

**Affiliations:** Department of Epidemiology and Public Health, University College London, London, UK; Department of Sociology, University of Vienna, Vienna, Austria; Department of Sociology, University of Vienna, Vienna, Austria; Department of Statistics, Computer Science, Applications, University of Florence, Florence, Italy

**Keywords:** COVID-19, Depression, Grandparenting, Life satisfaction, Well-being

## Abstract

**Objectives:**

Policies aiming at reducing rates of hospitalization and death from coronavirus disease 2019 (COVID-19) encouraged older people to reduce physical interactions. In England, until July 2021, provision of care for grandchildren was allowed only under very limited circumstances. Evidence also suggests that reduced face-to-face interactions took a toll on mental health during the pandemic. This study aims to investigate associations between changes in grandchild care provision during the first 8/9 months of the pandemic and grandparents’ mental health.

**Methods:**

Using prepandemic data from Wave 9 (2018/2019) and the second COVID-19 substudy (November/December 2020) of the English Longitudinal Study of Ageing, we first describe changes in grandchild care provision during the pandemic to then investigate, using regression models, associations between changes in grandchild care provision and mental health (depression, quality of life, life satisfaction), while controlling for prepandemic levels of the outcome variables.

**Results:**

About 10% of grandparents stopped looking after grandchildren altogether during the first 9 months of the pandemic, with 22% reporting an overall decrease in the amount of grandchild care provided. Compared to grandparents who mostly maintained unchanged their grandchild care provision, those who stopped altogether and those who mostly reduced the amount of grandchild care provided were more likely to report poorer mental health, even accounting for prepandemic health.

**Discussion:**

While measures to limit physical contact and shield older people were necessary to reduce the spread of COVID-19, policymakers should acknowledge potential adverse consequences for mental health among grandparents who experienced changes in their roles as grandchild caregivers.

Early in the coronavirus disease 2019 (COVID-19) pandemic, it was shown that risks of serious illness and death deriving from COVID-19 increased with age and that many preexisting diseases, also strongly correlated with age, increased morbidity and mortality risks (Jordan et al., 2020). Therefore, when governments enacted drastic public health measures to slow the spread of the virus and help prevent health services from being overrun (including lockdowns, shielding, and stay-at-home orders), there were clear recommendations that particularly older people should stay indoors, limit their travels and movements, as well as limit physical interactions with others (Ayalon, 2020; Perra, 2021). The implementation of such policies also meant the (temporary) suspension of grandchild care provision for many grandparents (Cantillon et al., 2021). However, the pandemic and associated policies restricting physical contact have also posed a risk to mental health, with ample evidence suggesting that the COVID-19 pandemic has resulted in poorer mental health (Patel et al., 2022; Santomauro et al., 2021).

Although around the world grandparents as providers of childcare are a common and significant form of intergenerational family support (Di Gessa et al., 2020; Hank & Buber, 2009; Hank et al., 2018; Ko & Hank, 2014; Zamberletti et al., 2018), and human relationships, intergenerational family contacts as well as the provision of grandchild care itself are important factors for mental health (Danielsbacka et al., 2022; Nyqvist et al., 2013; Umberson & Montez, 2010), to date no studies have investigated associations between changes in grandparenting and mental health during the time of the COVID-19 pandemic. The present study, therefore, aims to provide new and robust evidence addressing this gap. Using data from the nationally representative English Longitudinal Study of Ageing (ELSA), we investigate the longitudinal associations between changes in the amount of time grandparents looked after grandchildren in the first 8/9 months of the pandemic and three important indicators of mental health (depression, life satisfaction, and quality of life), while accounting for socioeconomic and demographic characteristics as well as prepandemic measures of mental health.

## Background

Grandparents vary in the extent to which they are involved in the lives of their grandchildren. Broadly speaking, grandparental childcare arrangements are distinguished in primary and secondary care. Primary carers are grandparents who assume legal responsibility for raising a grandchild. Whereas in the United States data are routinely collected on whether grandparents are primary carers, in Europe such information is generally inferred from living arrangements (Baker & Silverstein, 2008; Nandy & Selwyn, 2012). Secondary care refers to care complementary to the parental one: grandparents look after their grandchildren without, however, replacing the parenting functions and tend to do so to help parents go to work or give them a break (Di Gessa et al., 2020). In this case, their support can vary from being regular to occasional or for special occasions, and its intensity can range from a few hours per year to several per day (Glaser et al., 2013).

A considerable body of work shows that before the COVID-19 pandemic, around the globe, grandparents were significant providers of *secondary* grandchild care although the definition of grandchild care and therefore its prevalence and intensity vary across studies and countries (Di Gessa et al., 2020; Hank & Buber, 2009; Hank et al., 2018; Ko & Hank, 2014; Zamberletti et al., 2018). For instance, figures prepandemic show that more than 50% of grandparents looked after grandchildren in England (Di Gessa et al., 2020) and 20% of Italian grandchildren aged 0–13 were looked after by grandparents almost daily when their parents are at work (Zamberletti et al., 2018).

Especially in the early phases of the pandemic, as age is one the most important factors in diminishing a person’s chances to survive the COVID-19, governments’ strategy around the world has notably focused on targeting older people who have been advised not only to stay indoors and limit their physical interactions with others but also to stay away from their grandchildren and from younger people (Ayalon, 2020; Perra, 2021). In most European countries, governments discouraged grandparents from looking after grandchildren, unless in cases of “extreme necessity” if parents could not care for their children. The Israeli Ministry of Defence, Mr Bennett, stated that “the single most important insight is to separate old people from young people. The single most lethal combination cocktail is when grandma meets her grandchild and hugs him” (Ayalon, 2020). Also, several pediatricians, epidemiologists, and virologists suggested that overall “to be safe, grandparents really shouldn’t be doing childcare” (Glazer, 2020).

In England, the focus of this study, as part of the COVID-19 measures imposed by the government at the start of the UK lockdown in March 2020, schools and childcare settings were closed to all but vulnerable children and essential workers. Grandparents were told not to look after their grandchildren amid the coronavirus outbreak, with young people urged by the health secretary Hancock not to “kill [their] gran.” Although some rules were relaxed in the early summer of 2020, new guidance was complex: grandparents could only meet up with and care for their grandchildren either if grandparents lived by themselves or if grandchildren lived with a lone parent; moreover, all parties had to agree not to meet anyone else to avoid any chance of cross-household transmission (forming what was known as a “support bubble”). From December 2020 and up until July 2021 when most restrictions were lifted in England, grandparents providing childcare no longer had to live by themselves to form a bubble with their grandchildren. Grandparents and grandchildren belonging to the same bubble could also be visited at their home and could stay overnight. However, grandparents could only form one bubble (with a set of grandchildren) until March 2021 and were encouraged to limit physical contact with other people as much as possible for their own and public health.

While it is important to recognize that the clear aim and main benefits of guidelines focusing on staying at home and physical distancing are to contain the spread of the disease and save lives, such measures also all have the potential to substantially affect the mental health of the population both directly and indirectly (e.g., Brooks et al. 2020; Pfefferbaum & North 2020). The restriction of physical interactions has clearly posed a risk to mental health, and research investigating mental health sequelae of the pandemic both among older people (disproportionately affected by shielding policies and stay-at-home advice) and in the general population has found substantial deterioration in mental health, especially among those with preexisting mental or physical health conditions and low social support (Armitage & Nellums, 2020; Arpino et al., 2021; Brooks et al., 2020; Daly et al., 2020; Di Gessa & Price, 2021, 2022; Pierce et al., 2020; Steptoe & Di Gessa, 2021). However, little is known so far on how grandparents changed their provision of grandchild care during the pandemic and whether such changes played a role for their mental health.

Although grandchild care provision per se might not explain the health differential observed between grandparents who look after grandchildren and those who do not (Danielsbacka et al., 2019), and factors such as intensity and health outcomes add to the complexity of the relationship, several studies have found that grandparents providing secondary grandchild care tend to report better health (Chen & Liu, 2012; Di Gessa et al., 2016a, 2016b; Hughes et al., 2007; Ku et al., 2013). Positive associations have often been discussed in light of the emotional rewards and gratification stemming from this activity; the closeness of the relationships with grandchildren; stronger family ties and greater family support; and what is, to some, the most valued and satisfying of family roles (Danielsbacka et al., 2022; Kivnick, 1982). Therefore, abrupt changes or disruptions in intergenerational relationships might have negative consequences for the mental health of grandparents, particularly among those who suddenly (have to) stop looking after grandchildren. Previous studies that have investigated the consequences to the emotional well-being of grandparents after the loss of contact with their grandchildren (e.g., because of divorce or migration) suggest that such loss of contact adversely affects grandparents’ mental health, including lowered life satisfaction and depression (L. A. Drew & Smith, 1999; L. M. Drew & Silverstein, 2007). Generally, this negative effect was found particularly when the loss was unexpected as well as when grandparents were unable to exert control over the situation and were longing for a get-together with their grandchildren (L. A. Drew & Smith, 1999; L. M. Drew & Silverstein, 2007). For instance, L. M. Drew & Silverstein (2007) found that, among those who had lost contact with their grandchildren, grandparents who did so because of a “sudden event” (such as death or illness of the adult child, geographical separation, and crises faced by the grandchild) had the largest increase in depression symptoms, particularly soon after the abrupt loss of contact. In addition to suffering from the lack of contact with their grandchildren, these grandparents also suffered from the loss of their grandparental role, the successful enactment of which has been linked to higher life satisfaction and well-being (Kivnick, 1982; Reitzes & Mutran, 2004). Although previous studies have suggested that nonphysical contacts during the lockdown and the pandemic played a key role in attenuating the negative effects of the stressful context of COVID-19 on mental health (Arpino et al., 2021), we still expect that grandparents who during the pandemic stopped looking after grandchildren altogether or experienced significant disruptions to their role as grandchild carers might have experienced negative mental health consequences, even or especially in the short term. This is in line with the concept of “ambiguous loss” (Boss, 2009) that, adapted to disruptions in the grandparent–grandchild relationship, theorizes that the unfulfilled expectations and continued hope of reunion with their grandchildren can be a source of distress and emotional pain for grandparents who are not able to see and spend time with their grandchildren for reasons beyond their control. This can be particularly important in times of COVID-19, when changes in life and reduction in face-to-face contacts were abrupt and had no foreseeable deadline.

In light of this, the present study aims to understand whether and to what extent disruptions in grandparent–grandchild relationships in the form of grandchild care provision were associated with grandparents’ mental health during the pandemic (namely, depression, life satisfaction, and quality of life). To our knowledge, our study is the first to take into account changes in grandparental childcare provision during the first 8/9 months of the pandemic in England—exploiting newly collected data (see below). Moreover, given that older people who stayed at home, shielded, and minimized face-to-face contacts are also disproportionately more likely to have had poorer health prior to the pandemic, in this study we account for prepandemic characteristics that could potentially lead to a higher risk of social isolation and therefore to deteriorating mental health. Finally, given the detrimental independent role of lack of nonphysical contacts and loneliness on mental health and well-being, we also accounted for these important factors to better understand how changes in grandparental childcare provision relate to grandparents’ mental health.

## Method

### Study Population

We used data from the ELSA (Banks et al., 2019), a longitudinal biennial survey representative of individuals aged 50 and older in private households. During the pandemic, ELSA members were invited to participate online or by Computer-Assisted Telephone Interviewing to two COVID-19 substudies in June/July 2020 and in November/December (75% response rate in both waves, 94% longitudinal response rate). For this study, we restricted our sample to 2,468 grandparents who participated in the second COVID-19 wave (when information on the provision of grandchild care throughout the pandemic was collected); with at least one grandchild aged 15 or younger (as questions on grandparenting were not asked to grandparents with older grandchildren); no missing data on the provision of grandchild care during the pandemic; and with available socioeconomic and health information in the most recent prepandemic data (Wave 9, collected in 2018/2019). Further details of the survey’s sampling frame and methodology can be found at www.elsa-project.ac.uk. ELSA was approved by the London Multicentre Research Ethics Committee (MREC/01/2/91), with the COVID-19 substudies approved by the University College London Research Ethics Committee. Informed consent was obtained from all participants. All data are available through the UK Data Service (SN 8688 and 5050).

### Main Measurements of Interest

#### Grandparental childcare provision

Information on the provision of grandchild care throughout the pandemic was only collected in the second COVID-19 substudy in November/December 2020. All grandparents with at least one grandchild aged 15 or younger were asked “Did you look after any of your grandchildren without their parents present before lockdown started (in February)?” Those who reported having looked after grandchildren were then asked whether the amount of care provided to grandchildren changed during the lockdown period (March to June 2020), with options including “it increased,” “it decreased,” “it stayed the same,” or “it stopped.” Similar questions were also asked about changes in grandchild care provision during the summer months (June, July, and August 2020), and during the months since schools reopened (September, October, and November/December 2020) compared to prepandemic levels. Based on the possible combinations of answers, we classified respondents into four broad categories distinguishing between (a) those who did not provide grandchild care prepandemic; (b) those who during the first 8/9 months of the pandemic stopped completely to look after grandchildren; (c) those who overall decreased or interrupted their grandchild care; and (d) those who reported mostly the same or an increase in the amount of time they looked after grandchildren. Because less than 3% of the sample (*N* = 73) reported increases in care in at least two periods (with only 23 grandparents reporting increases in grandchild care provision throughout the whole period under study), it was not possible to consider those who increased care as a separate category. Full details about all possible patterns of changes and how grandparents were classified can be found in Supplementary Table S1.

#### Mental health

We considered three outcome measures of mental health assessed at the second COVID-19 substudy (November/December 2020): depressive symptoms, quality of life, and life satisfaction. Symptoms of depression were measured by an abbreviated eight-item version of the validated Center for Epidemiological Studies—Depression (CES-D) scale. The CES-D scale is not a diagnostic instrument for clinical depression but can be used to identify people “at risk” of depression in population-based studies (Radloff, 1977). This short version has good internal consistency (Cronbach’s α > 0.95) and comparable psychometric properties, validity, and reliability to the full 20-item CES-D (Karim et al., 2015). The scale includes eight binary (no/yes) questions that enquire about whether respondents experienced any depressive symptoms, such as feeling sad or having restless sleep, in the week prior to the interview. As done in previous studies, we classified respondents who reported four or more depressive symptoms on the CES-D scale as with elevated depressive symptoms (Turvey et al., 1999; Zivin et al., 2010). Furthermore, we considered subjective quality of life evaluated using the 12-item Control, Autonomy, Self-realization and Pleasure (CASP-12) scale. This is an abbreviated measure of the validated CASP-19 scale which was specifically designed for individuals in later life and used in a wide variety of ageing surveys (Hyde et al., 2003). CASP-12 contains 12 Likert-scaled questions measuring older people’s control and autonomy as well as self-realization through pleasurable activities. The possible range of CASP-12 scores is from 0 to 36, with higher scores indicating greater well-being; CASP-12 is treated as a continuous variable. Finally, we considered life satisfaction as a measure of personal well-being assessed using the Office for National Statistics well-being scale (“On a scale of 0 to 10, where 0 is ‘not at all’ and 10 is ‘very’, how satisfied are you with your life nowadays?”). This allows respondents to integrate and weigh various life domains the way they choose (Pavot & Diener, 1993).

#### Controls

Several potential confounders, known to be associated with grandparental childcare provision as well as with the main dependent variables, were adjusted for in all multivariable analyses. We controlled for age and age squared to account for nonlinear relationships with the outcome variables; sex; and ethnicity (White vs non-White participants due to data constraints in ELSA). To capture respondents’ socioeconomic characteristics, we controlled for prepandemic education, income, wealth, housing tenure, and paid employment (the latter during the pandemic). Educational level was recoded into low (below secondary), middle, and high (university or above) following the International Standard Classification of Education (http://www.uis.unesco.org/). We categorized respondents by quintiles of wealth (total net nonpension nonhousing wealth) and accounted for their equivalized total income (from paid work, state benefits, pensions, and assets). Housing tenure distinguished outright owners, owners with a mortgage, and nonowners. Paid employment distinguished retired, in paid work and not working from home, in paid work and mostly working from home, furloughed, and other (including homemakers, unemployed, and sick or disabled).

We also accounted for prepandemic health and controlled for disability (having impairments with basic and instrumental activities of daily living) and clinical vulnerability to COVID-19 (defined irrespective of age as reporting chronic lung disease, asthma, coronary heart disease, Parkinson’s disease, multiple sclerosis, diabetes; weakened immune system as a result of cancer treatment in the previous 2 years; body mass index of 40 or above; or having been advised to shield by their general practitioner/National Health Service; Di Gessa & Price, 2021). We further controlled for prepandemic measures of mental health (see above for derivation). Moreover, we included household composition, distinguishing between respondents living alone, with a partner only, with partner and child(ren), with child(ren) but not the partner, and any other arrangements. Furthermore, we controlled for whether respondents had any friends or family members who were hospitalized or died because of COVID-19, as this could be a proxy of heightened perceived fear of COVID-19 which might affect both mental health and the likelihood of wanting to mix with family members and friends outside the household. In robustness checks, we additionally controlled for nonphysical social contacts and loneliness during the pandemic. For nonphysical social contacts, we categorized respondents as having infrequent contact if they reported real-time contact (by telephone or video calling) less than once a week or never throughout the pandemic (= 0 otherwise). We distinguished between contacts with family outside the household and those with friends, although for the former it was not possible to consider the family members with whom they had contacts. We also considered whether respondents reported loneliness throughout the pandemic or not, where loneliness was assessed using the short version of the revised University of California Los Angeles (UCLA) loneliness scale (from 0 to 9, with scores of 6 and higher indicating greater loneliness).

Finally, as family structures have been associated with the provision of grandparental childcare (Di Gessa et al., 2016; Hank & Buber, 2009; Igel & Szyklik, 2011), we included the following grandchildren’s characteristics: total number of grandchildren; time to travel to their nearest grandchild (living in the same household or less than 15 min away; between 15 and 30 min away; more than 30 min away); and age of the youngest grandchild distinguishing between 0–2, 3–5, and 6–15 years. However, it is worth mentioning that these covariates were obtained from data collected in Wave 9, and that the distance to the closest grandchild and the age of the youngest one do not necessarily refer to the grandchild grandparents were looking after before the pandemic.

### Statistical Analysis

Following descriptive analysis, we investigated the longitudinal associations between patterns of grandparental childcare provision and mental health using logistic or linear models depending on the outcome. For all analyses, we present fully adjusted results accounting for sociodemographic, economic, and health covariates (including prepandemic relevant mental health measure) and grandchildren’s characteristics. We implement the so-called conditioning approach in which we control for covariates measured before the explanatory variables allowing to partially address confounding due to time-invariant unobservables and reverse causality. All analyses were performed using Stata 16. Sampling weights were employed to account for different probabilities of being included in the sample and for nonresponse to the survey.

## Results

### Descriptive Statistics

Table 1 shows socioeconomic, health, and demographic characteristics of grandparents with at least one grandchild aged 15 and younger by whether they were providing grandchild care prepandemic. Overall, 52% of grandparents were looking after grandchildren in February 2020. Grandparents who provided any grandchild care prepandemic were more likely to be younger, female, and to report better physical health. Also, a higher percentage of grandparents who did not provide prepandemic care reported infrequent contacts with friends and family during the first 8/9 months of the pandemic.

**Table 1.** Percent Distribution (*N*) of Sociodemographic, Economic, Health, and Social Contact Characteristics, by Prepandemic Grandchild Care Provision

	No childcare	Childcare	Total	*p* Value
Prepandemic sociodemographic and economic characteristics (*unless specified*)				
Mean age *during pandemic* (*SD*)	70.3 (9.3)	67.1 (7.2)	68.7 (8.40)	<.001
Female	52.7 (642)	60.3 (786)	56.4 (1,428)	.003
Non-White	6.5 (49)	5.6 (38)	5.9 (87)	.603
High educational qualification	18.5 (297)	18.3 (317)	18.2 (614)	.001
Middle educational qualification	48.1 (585)	56.2 (697)	51.9 (1,282)	
Low educational qualification	33.5 (306)	25.6 (266)	29.9 (572)	
Highest wealth quintile	20.0 (296)	21.9 (351)	21.0 (647)	.166
Second wealth quintile	18.4 (275)	22.5 (335)	20.6 (610)	
Third wealth quintile	19.9 (248)	18.2 (245)	19.0 (493)	
Fourth wealth quintile	18.4 (275)	22.5 (335)	17.4 (378)	
Lowest wealth quintile	22.9 (166)	21.4 (174)	22.1 (340)	
Employment status *during pandemic*				
Retired	68.4 (952)	58.9 (956)	63.4 (1,908)	.004
Employed (working not from home)	15.4 (96)	18.8 (139)	17.2 (235)	
Employed (working from home)	6.6 (60)	9.0 (87)	7.8 (147)	
Furloughed	1.8 (19)	4.8 (34)	3.3 (53)	
Sick/disabled/unemployed/other	7.8 (61)	8.6 (64)	8.6 (125)	
Home owner (outright)	66.3 (948)	70.7 (1,051)	68.6 (1,999)	.038
Home owner (with mortgage)	12.6 (88)	14.2 (105)	13.4 (193)	
Home renter	21.1 (152)	15.1 (124)	18.0 (276)	
Has had friends or relatives hospitalized or dead *during pandemic* because of COVID-19	11.5 (121)	13.3 (129)	12.4 (250)	.351
Prepandemic health characteristics				
Four or more CES-D depressive symptoms	14.3 (123)	13.4 (125)	13.8 (248)	.416
Mean CASP-12 (*SD*)	25.8 (6.67)	26.7 (6.21)	26.3 (6.48)	.014
Mean life satisfaction (*SD*)	7.5 (2.29)	7.5 (2.18)	7.5 (2.24)	.544
Clinically vulnerable to COVID-19	44.9 (507)	37.8 (453)	41.2 (960)	.005
With ADL or IADL limitations	27.6 (288)	21.2 (226)	24.3 (514)	.007
Social isolation and support *during pandemic*				
Living alone	24.2 (296)	21.2 (256)	22.6 (552)	.133
Living with partner/spouse only	52.9 (710)	59.6 (851)	56.4 (1,561)	
Living with partner/spouse and child(ren)	13.8 (103)	10.2 (98)	12.0 (201)	
Living with child(ren) but no partner/spouse	6.2 (51)	5.6 (37)	5.9 (88)	
Any other living arrangement	2.9 (28)	3.3 (38)	3.1 (66)	
Infrequent nonphysical contacts with friends	10.4 (115)	6.5 (92)	8.4 (207)	.002
Infrequent nonphysical contacts with family	6.7 (62)	3.6 (38)	5.1 (100)	.016
High UCLA loneliness	16.9 (174)	15.9 (170)	16.4 (344)	.607
Total number of respondents (*N*)	1,188	1,280	2,468	

*Notes*: ADL = activities of daily living; CASP-12 = 12-item Control, Autonomy, Self-realization and Pleasure; CES-D = Center for Epidemiological Studies—Depression; COVID-19 = coronavirus disease 2019; ELSA = English Longitudinal Study of Ageing; IADL = instrumental activities of daily living; *SD* = standard deviation; UCLA = University of California Los Angeles. Sample restricted to grandparents with at least one grandchild aged 15 or younger. Weighted data.

*Source*: ELSA, Wave 9 (2018–2019) and COVID-19 substudy Wave 2 (November/December 2020).

**Figure 1.**
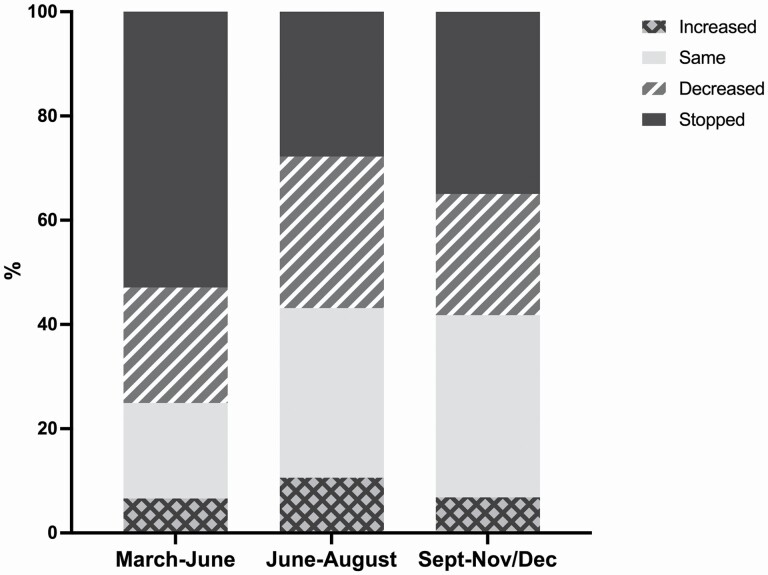
Changes over time in the amount of grandparental childcare provision compared to prepandemic levels. *Source*: ELSA, COVID-19 substudy Wave 2 (November/December 2020). Weighted data. Analyses restricted to grandparents with grandchildren aged 15 or younger who provided care prepandemic in February 2020. COVID-19 = coronavirus disease 2019; ELSA = English Longitudinal Study of Ageing.

Figure 1 shows that overall, among grandparents who provided grandchild care prepandemic, the percentage of ELSA grandparents who completely stopped looking after them was highest during the initial months of the pandemic (53%) when a national lockdown was announced and people were advised to stay at home as much as possible and to minimize contacts outside of their households. However, even during the summer of 2020 when some restrictions were lifted, more than a quarter of grandparents (28%) reported stopping caring for their grandchildren, with a higher percentage in the autumn (38%) when infection rates in England increased again and new restrictions were introduced. Similarly, between 20% and 30% of grandparents reported that the amount of care they provided for their grandchildren decreased compared to prepandemic. Grandparents who reported that the amount of grandchild care provided increased during the pandemic were around 6%–7% when restrictions were in place (spring and autumn 2020) and about 10% over the summer.

Table 2 shows the patterns of grandparental childcare over the first 8/9 months of the pandemic. Just less than half of the grandparents reported that they were not looking after grandchildren in February 2020 (before the pandemic). About one in five grandparents reported that the time spent looking after grandchildren was mostly the same or increased compared to prepandemic levels, whereas 22.4% reported that their engagement in grandchild care was mostly reduced or interrupted, and 9.5% completely stopped caring for their grandchildren.

**Table 2.** Distribution of Patterns of Grandparental Childcare Provision Across Three Time Points (March–June 2020, June–August 2020, and September–November/December 2020) and Unadjusted Mental Health by Patterns of Grandparental Childcare Provision

	*N*	%	% Elevated depressive symptoms (CES-D)	Mean quality of life (CASP-12)	Mean life satisfaction
No grandchild care prepandemic	1,188	47.8	29.7	24.58	6.85
Mostly same or increased	456	20.3	25.9	25.82	7.05
Mostly decreased or interrupted	568	22.4	27.2	25.37	6.91
Completely stopped	256	9.5	34.3	24.58	6.65
*N* respondents	2,468	100	28.9	25.02	6.88

*Notes*: CASP-12 = 12-item Control, Autonomy, Self-realization and Pleasure; CES-D = Center for Epidemiological Studies—Depression; COVID-19 = coronavirus disease 2019; ELSA = English Longitudinal Study of Ageing. Weighted data. A more comprehensive table with detailed patterns of changes in grandchild care provision can be found in Supplementary Table S1.

*Source*: ELSA, COVID-19 substudy Wave 2 (November/December 2020).

Table 2 also shows that mental health measured in the second wave of the COVID-19 ELSA substudy showed substantial variation by patterns of grandparental childcare. Respondents who stopped completely looking after grandchildren reported the highest percentages of elevated depressive symptoms (34.3%), the lowest mean life satisfaction (6.65), and the lowest quality of life among grandparents who looked after grandchildren prepandemic (mean CASP-12 = 24.58). Grandparents who overall had the same or increased levels of engagement in childcare, however, reported the best mental health (with 25.9% reporting elevated depressive symptoms; mean CASP-12 quality of life 25.82; and mean life satisfaction 7.05).

### Multivariable Analyses

To investigate how patterns of grandchild care provision during the pandemic were associated with mental health, we used logistic and linear regressions, depending on the outcome variable. We present (Table 3) results for the main variable of interest from fully adjusted models (full results of the fully adjusted models are in Supplementary Table S2). Accounting for sociodemographic and economic characteristics as well as for the health profile of the grandparents and their household composition, we found significant differences in mental health by patterns of changes in the provision of grandparental childcare. In particular, compared to grandparents who broadly maintained unchanged or increased grandchild care provision during the pandemic, grandparents who stopped completely looking after grandchildren were more likely to report high depressive symptoms (odds ratio [OR] = 2.04; 95% confidence interval [CI] = 1.30 to 3.19), lower levels of quality of life (*B* = −1.39, 95% CI = −2.26 to −0.52), and lower life satisfaction (*B* = −0.44; 95% CI = −0.83 to −0.06). Analyses also suggest that compared to those who maintained grandchild care during the pandemic, also those who mostly reduced or interrupted their provision of childcare had lower levels of quality of life (*B* = −0.82; 95% CI = −1.50 to −0.15) and lower life satisfaction (*B* = −0.32; 95% CI = −0.61 to −0.03). The direction of the association is similar also for decrease in grandchild care and depression, although this was not statistically significant. Compared to grandparents who did not look after grandchildren before the pandemic, those who kept the same amount of care reported better mental health (see Supplementary Table S3).

**Table 3.** Associations Between Grandparental Childcare Patterns and Mental Health

	Elevated depressive symptoms (CES-D)	Quality of life (CASP-12)	Life satisfaction
No grandchild care prepandemic	1.45* [1.01, 2.09]	−0.824** [−1.44, −0.21]	−0.232 [−0.51, 0.04]
Mostly same or increased	Ref	Ref	Ref
Mostly decreased or interrupted	1.25 [0.83, 1.89]	−0.823* [−1.50, −0.15]	−0.318* [−0.61, −0.03]
Completely stopped	2.04** [1.30 ,3.19]	−1.389** [−2.26, −0.52]	−0.441* [−0.83, −0.06]
*N* respondents	2,429	2,299	2,267

*Notes*: CASP-12 = 12-item Control, Autonomy, Self-realization and Pleasure; CES-D = Center for Epidemiological Studies—Depression; COVID-19 = coronavirus disease 2019; ELSA = English Longitudinal Study of Ageing. Fully adjusted models adjusted for age, age squared, sex, ethnicity, education, income, wealth, home tenure, employment, prepandemic disability, clinical vulnerability to COVID-19, relevant prepandemic mental health variable, household composition, number of grandchildren, distance to the closest grandchild, age of the youngest grandchild, as well as an indicator of whether friends and family members got hospitalized or died of COVID-19. Odds ratios [and 95% confidence intervals (CIs)] reported for elevated depressive symptoms, and beta coefficients (and 95% CIs) for the continuous outcome variables “Quality of life” and “Life Satisfaction”. For both continuous outcomes, the relevant health questions in Wave 9 were asked in the self-completion questionnaire (hence, the smaller sample size). Analyses are restricted to grandparents who reported grandparental childcare prepandemic. Weighted data. Detailed models can be found in the Supplementary Tables S2. Results from fully adjusted logistic and linear regression models.

*p < .05. **p < .01.

*Source*: ELSA, COVID-19 substudy Wave 2 (November/December 2020) and Wave 9 (2018/2019).

### Robustness Checks

Given that the category of grandparents who mostly decreased or interrupted their childcare provision is heterogeneous as it also includes some grandparents who, for one of the three time points considered, reported the same or increased levels of childcare provision, we conducted some checks to test the robustness of our results. In particular, we used a stricter definition of “decrease/interruption” of grandchild care provision, and further distinguished grandparents who reported that their amount of care either “decreased” or “stopped” *at all three periods considered*. The results obtained with this classification of changes in grandparental childcare (see Supplementary Table S4) suggest that it is those who “only” decreased or interrupted childcare throughout the pandemic who experienced worse mental health compared to grandparents whose amount of care broadly remained unchanged. However, these two categories of “decrease/interruption” are not significantly different from each other, and the weaker strength of associations found for those who “mostly decreased or interrupted” care might be due to the smaller sample size of this category. Also, given that stopping or reducing grandchild care could increase loneliness and reduce nonphysical contacts (particularly with children), potentially leading to poorer mental health, we repeated analyses including these variables in the regression models to rule out these potential mechanisms. As shown in Supplementary Table S5, results are consistent with the main models, with grandparents who stopped completely or mostly decreased caring for their grandchildren reporting poorer mental health, even when loneliness and infrequent contacts with friends and family are accounted for.

## Discussion

Providing grandparental childcare was quite common prepandemic. However, during the pandemic, grandparents were strongly advised to stop looking after grandchildren and more generally to limit their physical interactions with younger people, despite the known positive impact that intergenerational relationships have on mental health. In this paper, therefore, we investigate whether changes in grandparental provision during the pandemic are associated with three measures of mental health, namely depression, quality of life, and life satisfaction among grandparents in England.

Our analyses showed that overall, compared to grandparents who maintained unchanged or increased grandchild care provision during the pandemic, those who stopped looking after grandchildren altogether during the first 8/9 months of the pandemic as well as those who mostly reduced their involvement in grandchild care reported generally poorer mental health. Therefore, this study supports the idea that abrupt interruptions of older people’s role in society and family (in this study, of grandparents’ roles as care providers) are associated with poorer mental health, even when demographic and socioeconomic characteristics and prior health are accounted for. This is in line with the “ambiguous loss” argument and studies that found that grandparents unable to see and spend time with their grandchildren for reasons beyond their control might get frustrated and distressed about it, with negative consequences for their mental health (L. A. Drew & Smith, 1999; L. M. Drew & Silverstein, 2007). The COVID-19 pandemic might have acted in a very similar way, given that for many grandparents the pandemic, over which they could not exert any control, meant stopping or disrupting the amount of time spent looking after grandchildren (because of the guidelines or personal/family fears to get together) without knowing when they could be able to spend time with their grandchildren the same way they used to prepandemic.

### Strengths and Limitations

We investigated associations between changes in grandparental childcare provision and grandparents’ mental health during the pandemic using a longitudinal approach. To our knowledge, this was the first study to investigate this issue, drawing strength from using longitudinal data from the nationally representative ELSA. Even controlling for demographic, socioeconomic factors, and prepandemic health, our analysis supports the idea that both stopping altogether and disruptions to grandchild care provision were negatively associated with grandparent’s mental health. This might relate to lack of control over this interruption of care (dictated by the pandemic itself and government responses that are largely beyond people’s control) combined with the uncertainty of not knowing how long until grandparents could spend time with grandchildren again.

Our contribution, however, should be considered in light of some limitations. First, as mentioned above, ELSA does not collect detailed information about the childcare provided to each grandchild, but rather asks a more generic question related to all grandchildren and changes in the amount of time spent looking after them. Moreover, although in our analyses we considered several grandchildren’s characteristics (such as the age of the youngest grandchild and where the nearest grandchild lives), this information was collected in the prepandemic Wave 9 and might not relate to the grandchild to which grandparents provide care. Second, although the intergenerational decision-making process is generally related to the opportunities and resources of all three generations (Price et al., 2018), ELSA only asked questions about why grandparents changed the amount of care provided during the lockdown period (March to June 2020). Almost 70% of those who kept on looking after grandchildren or increased the amount of care provided during those first months of the pandemic reported doing so to help parents while working, whereas those who stopped or reduced grandchild care provision mentioned either that it was not practical (55%) or that childcare was not needed (43%). Although these give us an indication of how grandchild care family arrangements, particularly during the pandemic, might have reflected both parents’ employment status as well as school closures and (lack of) availability of formal childcare (Bordone et al., 2016; Di Gessa et al., 2016; Igel & Szyklik, 2011), ELSA does not collect any such information. Third, although in our analyses we controlled for whether grandparents had relatives or friends severely affected by COVID-19, ELSA did not collect information about respondents’ perception on individuals’ ability to tolerate and cope with the uncertainty due to COVID-19, or personality characteristics such as degree of risk tolerance or harm avoidance. These factors might help further understand both different behaviors and choices around levels of and changes in grandparental childcare and their subsequent relationship with mental health. Fourth, although we controlled for nonphysical social interactions during the pandemic, we did not have any specific measure on nonphysical contacts specifically with grandchildren. Fifth, as with all longitudinal surveys, ELSA also suffers from nonrandom cumulative attrition, with participants interviewed during the COVID-19 waves being more socioeconomically advantaged and having better health (based on prepandemic data) than those who were not. This is an unavoidable problem in longitudinal studies that was only partially corrected by using longitudinal weights in the analysis. Moreover, in our analyses, we could not control for other important changes that occurred during the pandemic (such as deterioration of physical health or living arrangements) and that are likely associated with both grandparental care and mental health. Finally, although ELSA is limited to the population of England, we believe that we can also draw inferences from this study for (at least) other European countries where policy recommendations and media advice were, in the early phases of the pandemic, quite similar and all pointed toward limiting as much as possible physical interactions between grandparents and grandchildren and where prepandemic engagement of grandparents in childcare were similarly high. If anything, we can expect that the negative associations observed between interruptions/disruptions of grandchild care and grandparents’ mental health would be larger in those countries (e.g., Mediterranean countries) where grandparental childcare is more normative, formal childcare services are limited, and grandparents tend to provide more regular grandchild care (Bordone et al., 2016; Di Gessa et al., 2016; Igel & Szyklik, 2011).

In summary, our study provides a picture of the broader consequences of the pandemic among a considerable proportion of older people, that is, grandparents who changed the amount of time they looked after grandchildren. Although recommendations to stop caring for grandchildren had the clear aim to save grandparents’ lives, it should be acknowledged that grandparents who stopped looking after grandchildren altogether and those who mostly reduced the amount of grandchild care provided were more likely to report poorer mental health. In emerging from the current pandemic and as restrictions are lifted, future studies should investigate whether the provision of grandparental childcare will resume prepandemic levels. Also, previous studies have often shown a rebound in emotional well-being after important demographic events including transition to parenthood, grandparenthood, or widowhood (Di Gessa et al., 2019; L. M. Drew & Silverstein, 2007; Peña-Longobardo et al., 2021), consistent with the findings of human resilience in the face of adverse events as well as the “set point” theory. Therefore, further research shall monitor grandparents’ mental health and well-being in the longer term, particularly for those who during the pandemic stopped looking after grandchildren altogether. If physical distancing policies remain a core strategy to protect individuals at higher risk from COVID-19 variants or indeed in a future pandemic, attention should be paid to addressing the mental health and wider needs of older people who may suffer from the loss of their roles.

## Supplementary Material

gbac104_suppl_Supplementary_Material

## Data Availability

The data were made available through the UK Data Archive. The English Longitudinal Study of Ageing was developed by a team of researchers based at University College London, NatCen Social Research, the Institute for Fiscal Studies, the University of Manchester, and the University of East Anglia. The data were collected by NatCen Social Research.
