# Supplementary material for: Changes in Grandparental Childcare During the Pandemic and Mental Health: Evidence From England
Source: J Gerontol B Psychol Sci Soc Sci. 2022 Sep 19;78(2):319–29. doi: 10.1093/geronb/gbac104 (PMC9494312; doi:10.1093/geronb/gbac104)
Supplement: gbac104_suppl_Supplementary_Material [file gbac104_suppl_supplementary_material.pdf]

Supplementary Table S1. Detailed patterns of changes in provision of grandchild care during the pandemic

Classification of changes in amount of childcare	Lockdown (March-June 2020)	Summer (June-August 2020)	Autumn (Sept-Nov/Dec 2020)	N	Unweighted percentage
Mostly same or increased	Same	Same	Same	123	4.98
	Same	Same	Stopped	8	0.32
	Same	Same	Decreased	13	0.53
	Same	Same	Increased	6	0.24
	Same	Stopped	Same	9	0.36
	Same	Stopped	Increased	4	0.16
	Same	Decreased	Same	9	0.36
	Same	Decreased	Increased	2	0.08
	Same	Increased	Same	8	0.32
	Same	Increased	Stopped	3	0.12
	Same	Increased	Decreased	4	0.16
	Same	Increased	Increased	1	0.04
	Increased	Increased	Increased	23	0.98
	Increased	Increased	Decreased	12	0.49
	Increased	Increased	Same	17	0.69
	Increased	Decreased	Same	3	0.12
	Increased	Stopped	Increased	2	0.08
	Increased	Same	Increased	2	0.08
	Increased	Same	Decreased	5	0.20
	Increased	Same	Stopped	1	0.04
	Increased	Same	Same	9	0.36
	Decreased	Increased	Increased	5	0.20
	Decreased	Increased	Same	11	0.45
	Decreased	Same	Increased	2	0.08
	Decreased	Same	Same	38	1.54
	Stopped	Increased	Increased	11	0.45
	Stopped	Increased	Same	18	0.73
	Stopped	Same	Increased	8	0.32
	Stopped	Same	Same	99	4.01
Mostly decreased or interrupted	Same	Decreased	Decreased	11	0.45
	Increased	Decreased	Decreased	3	0.12
	Same	Decreased	Stopped	2	0.08
	Same	Stopped	Decreased	2	0.08
	Same	Stopped	Stopped	8	0.32
	Increased	Stopped	Stopped	1	0.04
	Decreased	Same	Decreased	25	1.01
	Decreased	Increased	Decreased	6	0.24
	Decreased	Same	Stopped	3	0.12
	Decreased	Increased	Stopped	1	0.04
	Decreased	Decreased	Same	27	1.10
	Decreased	Decreased	Increased	3	0.12
	Decreased	Decreased	Decreased	105	4.25
	Decreased	Decreased	Stopped	18	0.73
	Decreased	Stopped	Same	3	0.12
	Decreased	Stopped	Increased	1	0.04
	Decreased	Stopped	Decreased	6	0.24
	Decreased	Stopped	Stopped	11	0.45
	Stopped	Same	Decreased	23	0.94
	Stopped	Increased	Decreased	9	0.36
	Stopped	Same	Stopped	7	0.28
	Stopped	Increased	Stopped	17	0.69
	Stopped	Decreased	Same	44	1.78
	Stopped	Decreased	Increased	10	0.41
	Stopped	Decreased	Decreased	74	3.00
	Stopped	Decreased	Stopped	66	2.67
	Stopped	Stopped	Same	39	1.58
	Stopped	Stopped	Increased	14	0.57
	Stopped	Stopped	Decreased	29	1.18
**Completely stopped**	Stopped	Stopped	Stopped	256	10.37
**No grandchild care pre-pandemic in Feb 2020**	--	--	--	1,188	48.14

Source: ELSA, COVID-19 sub-study Wave 2 (November/December 2020). Columns 2-4 specify whether the respondents reported increases, decreases, no changes, or interruption of grandchild care provision at each time point. Unweighted data

Supplementary Table S2. Associations between grandparental childcare patterns and mental health and well-being. Fully-adjusted regression models.

	Elevated depressive symptoms (CES-D)	Quality of life (CASP-12)	Life satisfaction
No grandchild care pre-pandemic	1.45* [1.01,2.09]	-0.824** [-1.44,-0.21]	-0.232 [-0.51,0.04]
Mostly same or increased	*Ref*	*Ref*	*Ref*
Mostly decreased or interrupted	1.25 [0.83,1.89]	-0.823* [-1.50,-0.15]	-0.318* [-0.61,-0.03]
Completely stopped	2.04** [1.30,3.19]	-1.389** [-2.26,-0.52]	-0.441* [-0.83,-0.06]
Female (Ref: Male)	1.78*** [1.36,2.32]	-1.057*** [-1.50,-0.61]	-0.412*** [-0.61,-0.22]
Age	0.97** [0.95,0.99]	0.039 [-0.00,0.08]	0.027** [0.01,0.05]
Age squared	1.00 [1.00,1.00]	-0.004* [-0.01,-0.00]	-0.000 [-0.00,0.00]
Non-White (Ref: White)	0.70 [0.35,1.41]	1.556* [0.09,3.02]	0.439 [-0.08,0.96]
Education (Ref: Low)			
Medium education	1.02 [0.74,1.40]	0.225 [-0.29,0.74]	0.231* [0.01,0.45]
Low education	1.00 [0.68,1.47]	0.722* [0.02,1.42]	0.394** [0.11,0.68]
Wealth (Ref: Lowest quintile)			
2nd lowest quintile	0.87 [0.56,1.35]	0.332 [-0.53,1.20]	-0.229 [-0.63,0.17]
3rd wealth quintile	0.82 [0.53,1.27]	0.322 [-0.59,1.23]	-0.084 [-0.47,0.30]
4th wealth quintile	0.63* [0.40,0.99]	0.456 [-0.46,1.37]	-0.052 [-0.44,0.34]
Highest wealth quintile	0.56* [0.34,0.91]	0.415 [-0.54,1.37]	-0.178 [-0.58,0.23]
Income	1.01 [0.94,1.08]	0.004 [-0.08,0.07]	-0.007 [-0.04,0.02]
Employment status (Ref: Retired)			
Employed not Working From Home (WFH)	0.39** [0.22,0.69]	1.425*** [0.65,2.20]	0.587*** [0.25,0.93]
Employed mostly WFH	1.50 [0.90,2.50]	0.788 [-0.12,1.69]	0.274 [-0.13,0.67]
Furloughed	1.12 [0.56,2.28]	0.451 [-1.41,2.31]	0.510 [-0.31,1.33]
Other employment	1.64 [0.94,2.84]	0.200 [-1.01,1.41]	0.195 [-0.34,0.73]
Housing (Ref: mortgage-free owner)			
Homeowner with mortgage	1.01 [0.63,1.62]	0.015 [-0.83,0.86]	-0.077 [-0.45,0.30]
Rented accommodation	0.69 [0.45,1.06]	0.341 [-0.52,1.20]	0.115 [-0.24,0.47]
Clinically vulnerable to COVID-19	1.21 [0.93,1.58]	-0.301 [-0.77,0.16]	0.003 [-0.21,0.21]
Disability	2.53*** [1.88,3.39]	-1.718*** [-2.39,-1.05]	-0.744*** [-1.02,-0.46]
Distance (Ref: ≤15m or cohabiting)			
Between 15 and 30m	1.33 [0.97,1.82]	-0.187 [-0.74,0.37]	-0.336** [-0.57,-0.10]
More than 30m	1.09 [0.79,1.49]	0.065 [-0.46,0.59]	-0.353** [-0.60,-0.11]
Number of grandchildren	1.02 [0.98,1.05]	0.045 [-0.02,0.11]	0.018 [-0.01,0.04]
Age youngest grandchild (Ref: 0-2)			
3-5	0.72 [0.51,1.02]	0.891** [0.26,1.52]	0.158 [-0.11,0.43]
6-15	0.76 [0.56,1.03]	0.250 [-0.31,0.81]	0.053 [-0.19,0.29]

Living arrangements (Ref: partnered)			
Living Alone	1.80*** [1.32,2.45]	-0.350 [-0.94,0.24]	-0.182 [-0.45,0.08]
Living w\ partner & children	1.08 [0.68,1.70]	-0.846* [-1.64,-0.05]	-0.226 [-0.55,0.10]
Single parent	3.07*** [1.67,5.64]	-1.094 [-2.44,0.26]	-0.340 [-0.89,0.21]
Other living arrangements	2.68* [1.26,5.68]	-1.992** [-3.44,-0.55]	-0.307 [-0.90,0.28]
Has had friends/relatives hospitalised or dead because of Covid-19	1.50* [1.04,2.17]	-0.205 [-0.99,0.58]	-0.269 [-0.59,0.05]
Pre-pandemic measure of relevant mental health/ well-being	5.34*** [3.60,7.90]	0.661*** [0.61,0.71]	0.402*** [0.34,0.46]
Constant		8.524*** [6.80,10.25]	4.422*** [3.73,5.12]
Observations	2,429	2,299	2,267

Sources: ELSA, COVID-19 sub-study Wave 2 (November/December 2020), COVID-19 sub-study Wave 1 (June/July 2020) and Wave 9 (2018/19).

Notes: Odds Ratios [and 95% Confidence Intervals (Cis)] reported for elevated depressive symptoms, and Beta coefficients [and 95% CIs] for the continuous outcome variables ‘Quality of life’ and ‘Life Satisfaction’. For both continuous outcomes, the relevant health questions in Wave 9 were asked in the self-completion questionnaire (hence the smaller sample size). Analyses are restricted to grandparents who reported grandparental childcare pre-pandemic.

+ $p < 0.10$* $p < 0.05$, ** $p < 0.01$, *** $p < 0.001$. Weighted data.

Supplementary Table S3. Associations between grandparental childcare patterns and mental health and well-being with 'no grandchild care pre-pandemic' as reference category. Fully-adjusted regression models.

	**Elevated depressive symptoms (CES-D)**	**Quality of life (CASP-12)**	**Life satisfaction**
No grandchild care pre-pandemic	*Ref*	*Ref*	*Ref*
Mostly same or increased	0.69*<br>[0.48,0.99]	0.824**<br>[0.21,1.44]	0.232^{+}<br>[-0.04,0.51]
Mostly decreased or interrupted	0.86<br>[0.60,1.24]	0.002<br>[-0.58,0.58]	-0.086<br>[-0.33,0.16]
Completely stopped	1.40^{+}<br>[0.95,2.06]	-0.566<br>[-1.31,0.18]	-0.209<br>[-0.55,0.13]
N respondents	2,429	2,299	2,267

Sources: ELSA, COVID-19 sub-study Wave 2 (November/December 2020) and Wave 9 (2018/19).
Notes: Fully-adjusted models adjusted for age, age squared, sex, ethnicity, education, income, wealth, home tenure, employment, pre-pandemic disability, clinical vulnerability to COVID-19, relevant pre-pandemic mental health variable, household composition, number of grandchildren, distance to the closest grandchild, age of the youngest grandchild, as well as an indicator of whether friends and family members got hospitalised or died of Covid-19.
Odds Ratios [and 95% confidence intervals (CIs)] reported for elevated depressive symptoms, and Beta coefficients [and 95% CIs] for the continuous outcome variables 'Quality of life' and 'Life Satisfaction'. For both continuous outcomes, the relevant health questions in Wave 9 were asked in the self-completion questionnaire (hence the smaller sample size). Analyses are restricted to grandparents who reported grandparental childcare pre-pandemic.
+ $p < 0.10$, * $p < 0.05$, ** $p < 0.01$, *** $p < 0.001$.

Supplementary Table S4. Robustness checks of the associations between grandparental childcare patterns and mental health and well-being using a stricter classification of 'decrease/interruption'. Fully-adjusted regression models.

	**% (N)**	**Elevated depressive symptoms (CES-D)**	**Quality of life (CASP-12)**	**Life satisfaction**
No grandchild care pre-pandemic	47.8 (1188)	1.45** [1.01,2.09]	-0.811** [-1.43,-0.19]	-0.226 [-0.50,0.05]
Mostly same or increased	*20.3 (456)*	*Ref*	*Ref*	*Ref*
Only decreased or interrupted	12.4 (309)	1.41 [0.89,2.24]	-0.918** [-1.74,-0.10]	-0.352** [-0.70,-0.01]
Mostly decreased or interrupted	10.0 (259)	1.09 [0.64,1.86]	-0.713+ [-1.53;0.11]	-0.271 [-0.59,0.06]
Completely stopped	9.5 (256)	2.05** [1.31,3.21]	-1.391** [-2.27,-0.51]	-0.440** [-0.83,-0.05]
N respondents	*2,468*	*2,429*	*2,299*	*2,267*

Sources: ELSA, COVID-19 sub-study Wave 2 (November/December 2020) and Wave 9 (2018/19).
Notes: Fully-adjusted models adjusted for age, age squared, sex, ethnicity, education, income, wealth, home tenure, employment, pre-pandemic disability, clinical vulnerability to COVID-19, relevant pre-pandemic mental health variable, household composition, number of grandchildren, distance to the closest grandchild, age of the youngest grandchild, as well as an indicator of whether friends and family members got hospitalised or died of Covid-19.
Odds Ratios [and 95% confidence intervals (CIs)] reported for elevated depressive symptoms, and Beta coefficients [and 95% CIs] for the continuous outcome variables 'Quality of life' and 'Life Satisfaction'. For both continuous outcomes, the relevant health questions in Wave 9 were asked in the self-completion questionnaire (hence the smaller sample size). Analyses are restricted to grandparents who reported grandparental childcare pre-pandemic.
+ $p < 0.10$, * $p < 0.05$, ** $p < 0.01$, *** $p < 0.001$.

Supplementary Table S5. Robustness checks of the associations between grandparental childcare patterns and mental health and well-being also considering social contacts and loneliness. Fully-adjusted regression models.

	**Elevated depressive symptoms (CES-D)**	**Quality of life (CASP-12)**	**Life satisfaction**
No grandchild care pre-pandemic	1.38^{+} [0.95,2.00]	-0.811** [-1.41,-0.22]	-0.222 [-0.50,0.06]
Mostly same or increased	*Ref*	*Ref*	*Ref*
Mostly decreased or interrupted	1.30 [0.85,1.98]	-0.851* [-1.50,-0.20]	-0.331* [-0.62,-0.04]
Completely stopped	1.97** [1.24,3.13]	-1.240** [-2.13,-0.35]	-0.377^{+} [-0.77,0.01]
N respondents	*2,416*	*2,287*	*2,255*

Sources: ELSA, COVID-19 sub-study Wave 2 (November/December 2020) and Wave 9 (2018/19).
Notes: Fully-adjusted models adjusted for age, age squared, sex, ethnicity, education, income, wealth, home tenure, employment, pre-pandemic disability, clinical vulnerability to COVID-19, relevant pre-pandemic mental health variable, household composition, number of grandchildren, distance to the closest grandchild, age of the youngest grandchild, an indicator of whether friends and family members got hospitalised or died of Covid-19, as well as social contacts and loneliness during the pandemic.
Odds Ratios [and 95% confidence intervals (CIs)] reported for elevated depressive symptoms, and Beta coefficients [and 95% CIs] for the continuous outcome variables ‘Quality of life’ and ‘Life Satisfaction’. For both continuous outcomes, the relevant health questions in Wave 9 were asked in the self-completion questionnaire (hence the smaller sample size). Analyses are restricted to grandparents who reported grandparental childcare pre-pandemic.
$+ p < 0.10$, $* p < 0.05$, $** p < 0.01$, $*** p < 0.001$.
